# A Web-Delivered, Clinician-Led Group Exercise Intervention for Older Adults With Type 2 Diabetes: Single-Arm Pre-Post Intervention

**DOI:** 10.2196/39800

**Published:** 2022-09-23

**Authors:** Morwenna Kirwan, Christine L Chiu, Thomas Laing, Noureen Chowdhury, Kylie Gwynne

**Affiliations:** 1 Faculty of Medicine, Health and Human Sciences Macquarie University Macquarie Park Australia; 2 Diabetes NSW & ACT Glebe Australia

**Keywords:** exercise, fitness, diabetic, physical activity, diabetes, functional fitness, community-based, older adult, videoconference, online, web-based, elder, geriatric, gerontology, balance, movement, internet-based, eHealth, digital health, weight, patient education, translational

## Abstract

**Background:**

The COVID-19 pandemic created unprecedented shifts in the way health programs and services are delivered. A national lockdown to prevent the spread of COVID-19 in Australia was introduced in March 2020. This lockdown included the closure of exercise clinics, fitness centers, and other community spaces, which, before the pandemic, were used to deliver Beat It. Beat It is an 8-week in-person, community-based, and clinician-led group exercise and education program for adults self-managing diabetes. To continue offering Beat It, it was adapted from an in-person program to a fully web-based supervised group exercise program for adults with type 2 diabetes (T2DM).

**Objective:**

This study aims to assess whether the *Beat It Online* program produced comparable health outcomes to the *Beat It* in-person program in terms of improving physical fitness (muscular strength and power, aerobic endurance, balance, and flexibility) and waist circumference in older adults with T2DM.

**Methods:**

Australians with T2DM who were aged ≥60 years were included. They were enrolled in *Beat It Online*, a twice-weekly supervised group exercise and education program conducted via videoconference over 8 weeks. Anthropometric measurements and physical fitness parameters were assessed at baseline and completion. The adaptations to *Beat It* are reported using the Model for Adaptation, Design, and Impact, including the type of changes (what, where, when, and for whom), the criteria for making those changes (why and how), and the intended and unintended outcomes. The intended outcomes were comparable functional fitness as well as physical and mental health improvements across demographics and socioeconomic status.

**Results:**

A total of 171 adults (mean 71, SD 5.6 years; n=54, 31.6% male) with T2DM were included in the study, with 40.4% (n=69) residing in lower socioeconomic areas. On the completion of the 8-week program, significant improvements in waist circumference, aerobic capacity, muscular strength, flexibility, and balance were observed in both male and female participants (all *P*<.001). The Model for Adaptation, Design, and Impact reports on 9 clinical, practical, and technical aspects of *Beat It* that were adapted for web-based delivery.

**Conclusions:**

This study found that *Beat It Online* was just as effective as the in-person program. This adapted program produced comparable health benefits across demographics and socioeconomic status. This study offers important findings for practitioners and policy makers seeking to maintain independence of older people with T2DM, reversing frailty and maximizing functional and physical fitness, while improving overall quality of life. *Beat It Online* offers a flexible and inclusive solution with significant physical and mental health benefits to individuals. Further evaluation of *Beat It* (both in-person and Online) adapted for culturally and linguistically diverse communities will provide greater insights into the efficacy of this promising program.

## Introduction

According to the International Diabetes Federation, more than half a billion people worldwide have diabetes [1], with type 2 diabetes mellitus (T2DM) accounting for 90% to 95% of cases [1,2]. The upward trajectory of diabetes prevalence has been described as a “pandemic with unprecedented magnitude spiraling out of control” [1].

Physical activity is a cornerstone of T2DM management, along with dietary and pharmacological interventions [3]. Current guidelines recommend that older adults with T2DM engage in at least 150 minutes per week of moderate-intensity aerobic exercise and perform moderate- or high-intensity resistance training on 2 or more days of the week [3]. Currently, two-thirds of older Australians do not meet these guidelines (irrespective of diabetes status) and are deemed insufficiently active to receive physical and mental health benefits [4]. Research has found that older adults who spend less time being physically active have lower overall functional fitness [5]. Functional fitness represents the physical capacity required to carry out typical daily activities independently, without the early onset of fatigue [6,7]. The typical decline in functional fitness with aging is known to lead to frailty, disability, morbidity, and mortality [7].

Physical activity has a substantial role in maintaining functional fitness [8]. Exercise, a structured form of physical activity, has been shown to increase physical fitness—strength, endurance, agility, balance, and flexibility—while also producing other physiological, psychological, and cognitive function benefits for adults with T2DM [3]. Physical fitness provides a protective factor for older adults with T2DM and reduces cardiovascular mortality, all-cause mortality, and the risk of falling [9-11]. Additionally, good physical fitness is known to extend years of active independent living while reducing disability and improving the quality of life [12]. Effective approaches to help older adults with T2DM improve physical fitness, which in turn will maintain functional fitness, are urgently needed.

Small and large-scale community-based supervised group exercise programs for older adults with T2DM have demonstrated effectiveness in improving physical fitness in the short term (immediately after intervention) [13-19], with follow-up studies demonstrating that these improvements can be maintained for up to 1 year [20-22]. Public health recommendations and governmental measures during the ongoing COVID-19 pandemic resulted in various restrictions on daily living including social distancing, isolation, and house confinement. Although these restrictions helped to abate the rate of infection [23], such limitations created considerable disruption to the routine supportive care of those managing T2DM [24]. Many health programs and providers had to pivot to delivering services over the web during this time. A national lockdown to prevent the spread of COVID-19 in Australia was introduced in March 2020. This lockdown included the closure of exercise clinics, fitness centers, and other community spaces, which, before the pandemic, were used to deliver *Beat It*, an 8-week in-person, community-based, and clinician-led group exercise and education program for adults self-managing diabetes. To continue offering this translational program, *Beat It* was adapted from an in-person program to a fully web-based supervised group exercise program for adults with T2DM.

The short- and long-term health and physical fitness outcomes from the traditionally delivered in-person *Beat It* program have been published elsewhere [19,22]. This research found that completing the *Beat It* program significantly improved physical fitness (muscular strength and power, aerobic endurance, balance, and flexibility) and reduced waist circumference for older adults (age ≥60 years), and these benefits were maintained for up to 12 months postprogram [22]. What is unclear is whether the adapted *Beat It* *Online* program provides comparable benefits to participants. Thus, the aim of this study was to assess whether the *Beat It Online* program was effective in improving physical fitness (muscular strength and power, aerobic endurance, balance, and flexibility) and waist circumference during the height of the COVID-19 pandemic.

## Methods

*Beat It Online* is an 8-week program that involves twice-weekly synchronous group exercise and education sessions, delivered via videoconferencing, by accredited exercise physiologists (AEPs).

### Program Design

Programs were delivered by AEPs to participants who resided in New South Wales (NSW) and the Australian Capital Territory (ACT). Exercise sessions include moderate-intensity aerobic, resistance, flexibility, and balance-based exercises, and the education sessions focus on different areas of diabetes self-management. Participants completed a pre-exercise screening, baseline measures, and fitness testing during a web-based 1-on-1 initial consultation with the AEP. This consultation also included motivational interviewing to ascertain participants’ goals and their facilitators and barriers to change [25].

The information gathered during the consultation was used to create a customized exercise program with the participant, considering their physical ability, existing fitness levels, and any comorbidities or injuries that could be exacerbated by exercise. Each exercise program comprised home exercise options that participants could replicate with minimal equipment or using items available around the home, including dynamic warm-up and cooldown, and aerobic (eg, walking, stepping, aerobics, and shadow boxing), resistance (eg, body weight, resistance bands, free weights, or household items of equivalent weight—for example, cans of food or bags of rice as hand weights), balance (eg, different standing balance variations), and flexibility exercises (eg, static stretching of major muscle groups). During the web-based group exercise sessions, participants completed their exercise program under the supervision of an AEP, who was able to modify exercises in accordance with the participant’s progress.

### Platform and Delivery

Zoom videoconferencing software (version 5.2.1; Zoom Video Communications) was the preferred platform for delivering the group exercise and education sessions. All AEPs purchased a subscription to Zoom as a security precaution to ensure that 2-factor authentication, passcodes, and waiting rooms were available; thus, incidents such as *Zoom bombing* and other security concerns were prevented.

### Facilitator Training

All AEPs completed a specialized facilitator training program called *Beat It Trainer* to ensure the consistent and effective delivery of the program. This accredited continuing professional development training course, certified by *Diabetes Qualified*, a subsidiary of Diabetes NSW & ACT, consisted of 12 hours of asynchronous web-based modules and assessments, followed by a 1-day synchronous session focused on the clinical, practical, and technical requirements of delivering the *Beat It* program. Additionally, AEPs also completed a supplemental 2-hour synchronous training session focused on considerations for web-based delivery. To maintain this certification, all AEPs are required to complete a refresher course within 2 years of completing the initial training.

### Recruitment

From May 2020 to October 2021, participants were recruited through email using the National Diabetes Services Scheme database or advertisements on the Diabetes NSW & ACT website. Prior to commencing, participants were required to provide evidence of medical clearance to exercise from their general practitioner and were then eligible to attend their web-based initial health and fitness assessment with their AEP. Standard exclusion criteria for the *Beat It Program* (in-person and Online) are outlined in Multimedia Appendix 1. Health and fitness assessments were performed at baseline and at the completion of the program. For inclusion into the current study, participants had to be aged ≥60 years, have a clinical diagnosis of T2DM, and have completed the web-based health and fitness assessment at baseline and at 8 weeks at the completion of the program.

### Ethics Approval

This study was approved by the Macquarie University Human Ethics Committee (5201950887424).

### Study Design and Measures

This study used a pre-post evaluation design where participants completed web-based physical assessment sessions at baseline and at 8 weeks of *Beat It Online*, as well as an evaluation questionnaire prior to and at the completion of the program. Sociodemographic information, including gender, date of birth, and residential postcode, was collected. Postcodes were used as a measure of socioeconomic status as previously described [19]. Height and weight were used to calculate BMI, and BMI was then categorized as healthy, overweight, and obese, according to the World Health Organization [26]. An assessment of upper and lower body muscular strength, aerobic capacity, flexibility, and balance were performed using the 30-second sit-to-stand test; 30-second seated arm curl test; 2-minute step test; seated sit-and-reach test; and 1-legged stand test, respectively [7]. The average of left and right sit-and-reach test and 1-legged stand test was used. Participants with missing data for gender, age, and waist circumference measures were excluded from analysis.

The Diabetes Empowerment Scale (DES) is a measure of psychosocial self-efficacy that was developed specifically for a diabetes population. The DES short form contains 8 items that can be used to provide a brief general assessment of diabetes psychosocial self-efficacy [27]. Participants are asked to indicate their agreement to 8 individual statements related to their ability to manage diabetes. Each item is rated on a 5-point scale: 5=strongly agree‚ 4=somewhat agree‚ 3=neutral‚ 2=somewhat disagree‚ and 1=strongly disagree. The overall score is the sum of the scores for each individual item, divided by the number of items (8 items).

The Patient Activation Measure (PAM) assesses a person’s self-reported knowledge, skill, and confidence for the self-management of their health or chronic condition [28]. Each item is rated on 4-point scale: from 1=strongly disagree to 4=strongly agree, with an additional “not applicable” option. The total PAM score is the sum of raw scores for each item, divided by the number of items excepting the nonapplicable items and then multiplied by 13. Higher scores are associated with the person taking action.

### Analysis

Data analysis was performed using SPSS statistical software (version 27; IBM Corp). Mean and SD were calculated for continuous variables. Frequencies and percentages were calculated for categorical variables, excluding participants with missing data for that variable. Paired 2-tailed *t* tests were performed to identify differences in fitness measures at baseline and program completion.

**Figure 1:**
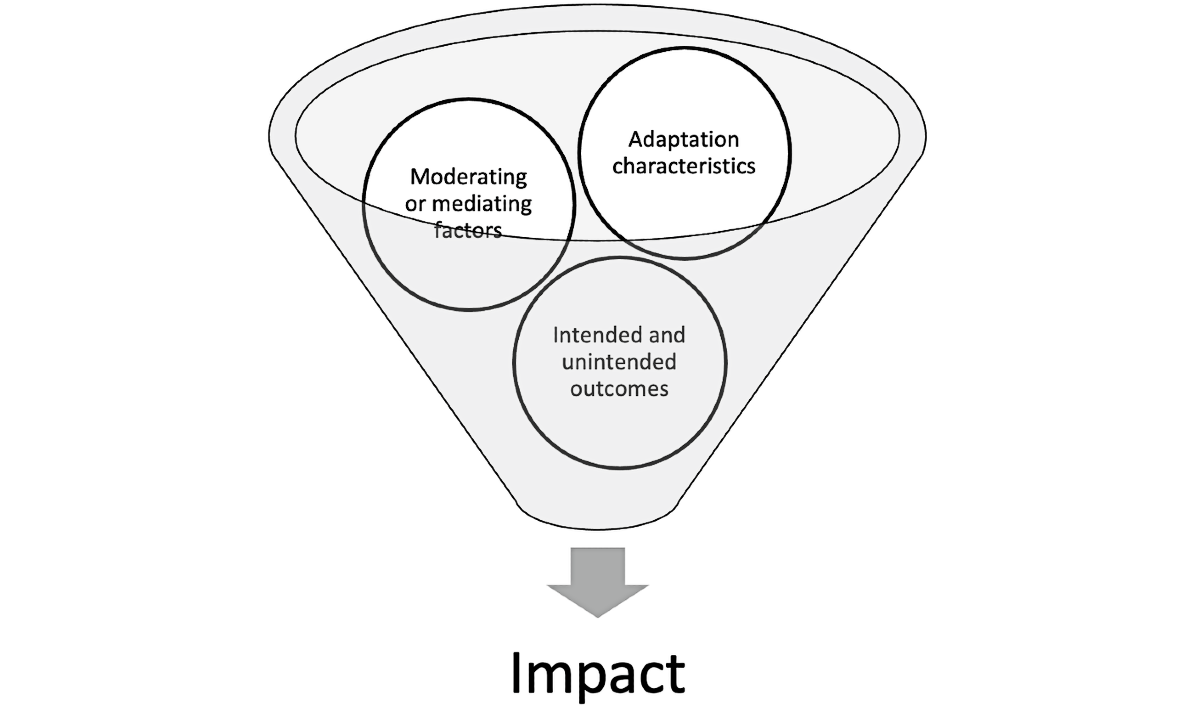
Model for Adaptation, Design, and Impact.

The adaptation of *Beat It* has been represented using the Model for Adaptation, Design, and Impact (MADI) [29]. This model has 3 domains: adaption characteristics (what was modified and how, for whom and when, and who was involved in decision making); moderating or mediating factors (adaptation aligned with the core elements of the intervention or implementation strategy, clear goals for adaptation, and adaptations implemented consistently); and intended and unintended outcomes. The analysis of the 3 domains together helps explain impact (Figure 1).

## Results

### Beat It MADI Results

The *Beat It Online* adaptations using the MADI model are shown in Table 1. There were 9 clinical, practical, and technical aspects of *Beat It* that were adapted for web-based delivery. Each of these aspects had moderating and mediating factors to support program fidelity while operationalizing *Beat It* in a web-based context. The intended outcomes were comparable functional fitness and physical and mental health improvements across demographics and socioeconomic status. These outcomes are reported in Table 2. At this stage, unintended outcomes are unknown and will be explored in a follow-up study. The impact was that *Beat It Online* was as effective as the in-person program.

**Table 1:** Model for Adaptation, Design, and Impact report of adaptations and mediating and moderating factors.

Adaptation areas	Mediating and moderating factors, program stage	
	Beat It (in-person)	Beat It Online
Facilitator training	12 hours web-based learning and 1-day in-person practical training	Additional 2 hours covering key considerations for web-based delivery
Marketing	Direct mail (via post), email, and website	Digital only—email and website
Participant resources	*Beat It* participant handbook, home exercise resource, and Theraband	Additional resources guiding participants on technical requirements and equipment required to access web-based sessions: internet connectivity and speed tests how to set-up or log in to videoconferencing platform participant pre–web-based session checklist outlining key safety considerations to be followed every session
Trainer resources	*Beat It* in-person delivery manual and *Beat It* facilitator manual (education sessions)	Additional content added to the *Beat It Online* delivery manual: assessing participant suitability additional Beat It Trainer requirements required equipment and technical capabilities, safety requirements, legal and professional practice requirements, and privacy and record keeping considerations web-based delivery considerations specific to exercise and education session delivery assisting Beat It trainers in selecting appropriate videoconferencing platforms ways to maintain communication and establish visual cues (eg, Zoom gallery view) to ensure participants are performing exercises confidently and safely trainer checklists and session guides to follow prior, during, and following each session
Medical clearance	Standardized medical clearance form, including recommended program inclusion or exclusion criteria, medical history, medications, and latest Hemoglobin A_1c_ and lipid test results; participants typically bring a physical copy of the medical clearance form to initial consultation with a *Beat It* trainer	Additional considerations and exclusion criteria for determining suitability to join the web-based program, including: client digital literacy client needs and goals risks including precautions and contraindications physical capacity of client to undertake session client ability to provide consent capacity to access technology need and availability for a client support person (eg, family, carer, and allied health assistant) to assist in consult and sessions further information relating to hypoglycemia frequency and falls risk requested from referring medical practitioner additional instructions relating to sending medical clearance information safely and securely via appropriate web-based methods (eg, encrypted email and fax) to the participant’s Beat It trainer
Preprogram	Preprogram resources sent including welcome letter confirming program registration, medical clearance, and initial consultation process and the *Beat It* trainer books initial assessment appointment	Additional resources guiding participants on what equipment is required to access *Beat It Online*, including: technical requirements (eg, internet connection, appropriate device for video calls, and active email address) initial assessment equipment (eg, measuring tape, weight scales, suitable chair, and hand weight or substitute to perform exercise tests from home) safety considerations (eg, appropriate exercise space, clothing, and access to blood glucose monitor and hypoglycemia treatment) provision of step-by-step guide of how to access the Beat It trainer’s selected videoconferencing platform at the time of assessment booking, the Beat It trainer also assessed participant’s technical proficiency with using the videoconferencing tool
Initial and final assessment	Conducted in person Obtain medical clearance, participant informed consent, and emergency contact information and complete prescreening questionnaire Complete baseline measurements, including height, weight, waist circumference, blood pressure, and heart rate Complete exercise tests including the 6-minute walk test, 30-second sit-to-stand test, 30-second seated arm curl test, seated sit-and-reach test, and 1-legged stand test Goal setting	Differences: both assessments conducted over the web informed consent form sent digitally further participant emergency contact information was collected, including physical address participant would be completing web-based exercise sessions from, presence of friend or family at this address during sessions, and education related to having access to blood glucose monitor and hypoglycemia treatment available during sessions baseline measurement protocols were adapted to support participants complete these themselves with guidance from a Beat It trainer via video (eg, using string or sewers tape to measure waist circumference) and requested from the referring general practitioner as part of medical clearance process (eg, resting blood pressure if participant does not have access to a blood pressure monitor) exercise testing protocols were modified to allow participant completion from home 6-minute walk test was replaced with a 2-minute step-in-place test to account for space constraints and allow participants to be monitored on camera household items were used to facilitate other tests (eg, 2-3 kg bag of rice for seated arm curl test and ruler used for seated sit-and-reach test)
Exercise sessions	Capped at 12 participants per session; in-person exercise sessions consist of a warm-up, followed by a combination of aerobic, resistance, balance, and flexibility exercises tailored to participants abilities, followed by a cooldown period	Differences: capped at 6 participants per session to enable adequate supervision in a web-based setting corresponding pre-exercise checklists for the Beat It trainer and participants detailing important steps the participants must take leading into each exercise session including ensuring technology is setup correctly allowing the sessions to be viewed clearly, confirming participants have hypoglycemia treatment available, ensuring their exercise area is free from obstructions, and asking participants to take pre- or postexercise blood glucose measurements guidelines on camera and microphone settings to ensure Beat It trainers can be seen and heard, and participants can be monitored effectively and the use of visual cues and telehealth functions to provide feedback and breaking up exercises to check-in with participants Beat It trainers to structure sessions by providing 3-4 different options for each exercise delivered to the group; these options include regressions and progressions for each exercise and ensure participants can complete a similar exercise at the same time, dependent on their ability Beat It trainers encouraged to keep sessions as creative, fun, and engaging as possible with ideas such as dress-up themes, activity-based challenges, and games to encourage social interaction among the group
Education sessions	6 x 30 min person-centered education sessions on various lifestyle and diabetes management topics delivered in person	Differences: delivered over the web used screen-sharing functions and web-based whiteboards to collate participant responses

**Table 2:** Summary of assessment data.

Assessment	Male					Female			
	n	Baseline, mean (SD)	Postprogram, mean (SD)	*P* value	n		Baseline, mean (SD)	Postprogram, mean (SD)	*P* value
Waist circumference (cm)	54	114.3 (12.5)	110.6 (12.5)	<.001	117		102.7 (14.3)	100 (13.3)	<.001
Seated sit-and-reach (cm)	46	–7.8 (11.1)	–4.3 (10.2)	<.001	106		–2.5 (11.9)	0.2 (11.8)	<.001
30-second sit-to-stand (reps)	51	13.2 (3.9)	16.2 (5.2)	<.001	116		12.6 (4.6)	15.7 (5.3)	<.001
1-legged stand test (s)	52	27.1 (20.3)	34.5 (18.5)	<.001	115		25.4 (19.9)	33.2 (20.5)	<.001
2-minute step test (reps)	45	68.5 (22.5)	87.9 (26.8)	<.001	105		69.2 (26.2)	86.8 (30.3)	<.001
Arm curl (reps)	50	20.1 (9)	26.0 (8.8)	<.001	114		18.9 (8.6)	23.5 (8.5)	<.001

### Beat It Online Participant Results

A total of 171 individuals were included in the study. These individuals were aged ≥60 years, had reported a diagnosis of T2DM, and had participated in *Beat It Online*. Of the 171 individuals, 54 (32%) were male, age ranged from 60 to 89 years with a mean age of 71 (SD 5.6) years, and 69 (40%) resided in lower socioeconomic areas. Participants attended between 5 and 16 exercise sessions, with 70% (n=119) of the cohort attending at least 14 out of the 16 sessions. For the education sessions, participants attended between 0 and 6 sessions, with 80% (n=137) of the cohort attending at least 5 out of the 6 education sessions.

**Figure 2:**
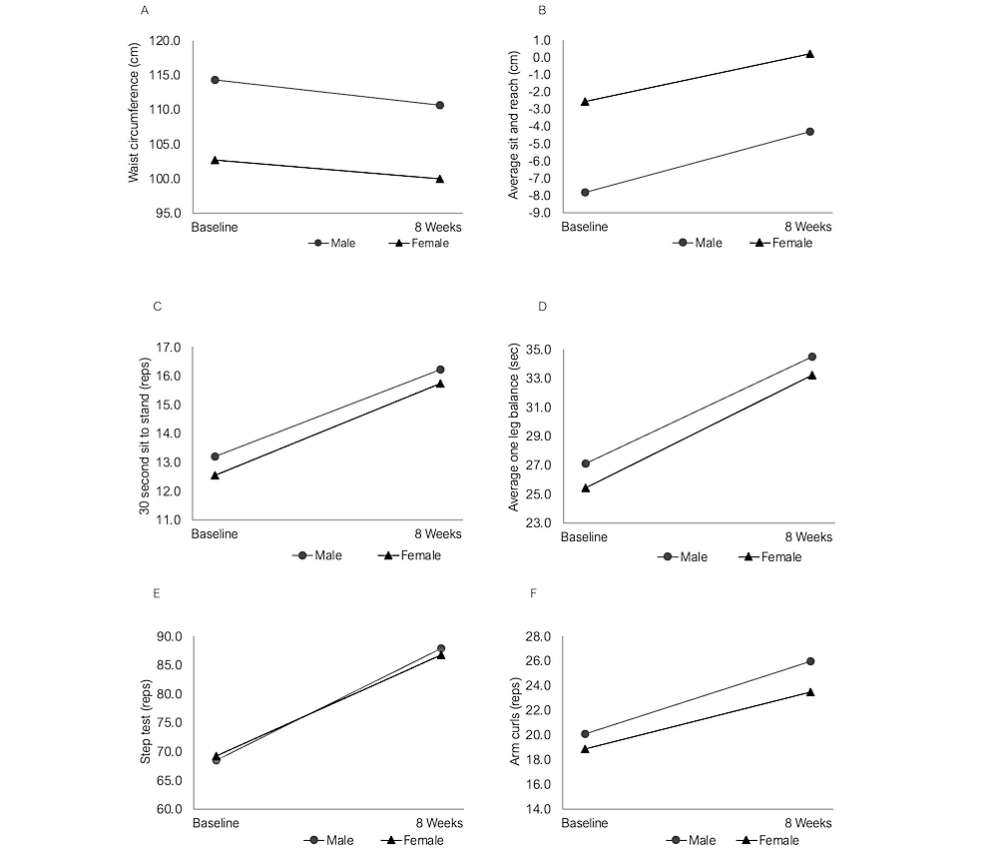
Mean fitness and health measures at baseline and at program completion, stratified by gender.

Improvements in waist circumference, aerobic capacity, muscular strength, flexibility, and balance were observed postprogram in both male and female participants (Table 2 and Figure 2).

Survey evaluation data was received from 49 (29%) of the 171 participants. Most participants rated their health as being good to excellent both at baseline and postprogram (n=38, 78% vs n=36, 74%), whereas improvements in their general quality of life rated as being good to excellent were reported postprogram (n=38, 78% vs n=47, 96%).

Significant improvements in DES scores were reported postprogram (3.9 vs 4.3; *P*<.001), whereas nonsignificant improvements in PAM scores were observed (44.3 vs 45.4; *P*=.07).

## Discussion

### Principal Findings

This study found that *Beat It Online* was just as effective as the in-person program [19,22]. This adapted program produced comparable health benefits, across demographics and socioeconomic status. The COVID-19 pandemic has necessitated limitations on physical interaction, requiring the rapid web-based adaptation of key health programs and services. This study found that the *Beat It Online* program, an 8-week clinician-led supervised group exercise program, significantly improved physical fitness (aerobic capacity, strength, balance, and flexibility), reduced waist circumference, and improved self-efficacy and quality of life in older adults with T2DM. This finding is important because globally, it has been reported that during the home confinement of lockdowns and necessary isolation, most individuals decreased their physical activity levels and increased sedentary behavior [30], and those with T2DM had increased body weight and worsened their glycemic control [31].

In this study, we used MADI, an implementation science framework, to transparently evaluate and report on the adaptations to the *Beat It* program [29]. We identified important adaptation characteristics, their intended and unintended impacts, and potential mediators and moderators of adaptations’ impact on outcomes. The adjustments made to the processes and protocols for *Beat It* were similar to that outlined by Schwartz and colleagues [32] in their pivot to delivering web-based, synchronous group exercise programs to older adults. It is important to not assume that an exercise specialist (or any health practitioner) who primarily delivers services in-person will be capable of delivering a comparable service over the web. Research has shown that the most prevalent barrier for the organizational adoption of telehealth, by a wide margin, is technically challenged health practitioners [33]. A comprehensive capability framework, such as the one developed by Davies and colleagues [34], provides important guidance on the core capabilities, curricula, and professional development needed in this space.

The focus in diabetes care has traditionally been around the optimization of glycemic control and deterrence of complications [35]. However, the prevention of frailty and improvement in physical function have now emerged as new targets of diabetes management [35]. This focus is mainly driven by the significant adverse impact that early onset frailty and declines in functional fitness have on health outcomes, including glycemic control, independence, and quality of life in people with T2DM [35]. The *Beat It Online* program produced significant improvements in all physical fitness measures, as well as self-efficacy and self-reported quality of life, in a cohort that was aged 71 (SD 5.6; range 60-89) years. At a time when older adults across the world were told to isolate at home, *Beat It Online* provided a socially supportive setting for vulnerable individuals with a chronic condition and comorbidities to exercise safely, boost their mood, and connect with their peers while following health directives related to COVID-19. This program is particularly important given that research has shown that stay-at-home orders have resulted in the loss of formal and incidental social connections, putting older adults at acute risk for loneliness [36]. Furthermore, participation in moderate to high volumes of physical activity during and following periods of COVID-19 containment has been associated with better mental health and well-being compared to inactive adults [37].

The *Beat It Online* program has debunked the pervasive stereotype that older adults are resistant and incapable of engaging with new technologies [38]. The participants in this study demonstrated that they were skilled at using videoconferencing software in a way that helped them improve their physical health and mental well-being. This openness to using technology for a clear purpose is well supported in the literature [39] and aligns directly with research showing that most older adults have a strong motivation to learn new skills and continue living fully through learning [40]. This finding has implications for practitioners and policy makers who may assume that this demographic only prefers in-person programs and services.

A limitation of this study is that it used a pre-post evaluation with no comparison group, which is a common design for translational community-based programs [41]. Another limitation is that we only evaluated the short-term effectiveness of the *Beat It Online* program. A longer follow-up is needed to ascertain if participants maintained the benefits gained from this program, any unintended outcomes, and whether *Beat It Online* is pragmatic outside of a global pandemic. We conducted a 1-year follow-up of the in-person *Beat It* program and concluded that participants maintained improvements in their health 12 months after completing the 8-week program [22].

### Conclusions

This study revealed that a fully web-delivered, clinician-led, and supervised group exercise program provided important health benefits to older adults with T2DM. This study offers important findings for practitioners and policy makers seeking to maintain independence of older persons with T2DM, reversing frailty and maximizing functional and physical fitness while improving overall quality of life. The COVID-19 pandemic has created unprecedented shifts in the way key health programs and services are delivered. *Beat It Online* offers a flexible and inclusive solution with significant physical and mental health benefits to individuals. Further evaluation of *Beat It* (both in-person and Online) adapted for culturally and linguistically diverse communities will provide greater insights into the efficacy of this promising program.

## Appendix

Multimedia Appendix 1 Standard exclusion criteria for Beat It (in-person) and Beat It Online.

## Abbreviations

ACT: Australian Capital Territory
AEP: accredited exercise physiologist
DES: Diabetes Empowerment Scale
MADI: Model for Adaptation, Design, and Impact
NSW: New South Wales
PAM: Patient Activation Measure
T2DM: type 2 diabetes mellitus
