# Supplementary material for: A Web-Delivered, Clinician-Led Group Exercise Intervention for Older Adults With Type 2 Diabetes: Single-Arm Pre-Post Intervention
Source: J Med Internet Res. 2022 Sep 23;24(9):e39800. doi: 10.2196/39800 (PMC9547336; doi:10.2196/39800)
Supplement: Multimedia Appendix 1 [file jmir_v24i9e39800_app1.docx]

**Multimedia Appendix 1: Standard exclusion criteria for Beat It (in-person) and Beat It Online**

- Unstable diabetes, including, but not limited to:
- HbA1c >11% (97mmol/mol)*
- More than 2 hypoglycaemia episodes a week*
- Asymptomatic hypoglycemia*
- (*unless cleared by an endocrinologist)
- Significant diabetes related complications
- Severe respiratory disease
- Unstable Angina
- < 6 months post Myocardial Infarction, CABG, valve replacement, post stent, or other cardiac surgery** (**unless medical clearance from a cardiologist has been received prior to participation)
- Severe uncontrolled metabolic disorders
- Severe neurological disorders
- Severe musculoskeletal conditions that would prohibit exercise
- Renal Failure
- Heart Failure
- Uncontrolled hypertension
- Resting blood pressure >180mmHg systolic and/or >110mmHg diastolic
- Acute systemic illness or fever
- Carotid artery stenosis (**unless cleared by a
- cardiologist)
- Pericarditis or myocarditis
- Any known aneurysm
- Significant cardiac dysrhythmias
